# Phosphorylation Sites Identified in the NEIL1 DNA Glycosylase Are Potential Targets for the JNK1 Kinase

**DOI:** 10.1371/journal.pone.0157860

**Published:** 2016-08-12

**Authors:** Aishwarya Prakash, Vy Bao Cao, Sylvie Doublié

**Affiliations:** 1 Department of Oncologic Sciences, Mitchell Cancer Institute, University of South Alabama, 1660 Springhill Avenue, Mobile, AL, 36604-1405, United States of America; 2 Department of Microbiology and Molecular Genetics, The Markey Center for Molecular Genetics, University of Vermont, Stafford Hall, 95 Carrigan Drive, Burlington, Vermont, 05405-0068, United States of America; University of Minnesota Twin Cities, UNITED STATES

## Abstract

The NEIL1 DNA glycosylase is one of eleven mammalian DNA glycosylases that partake in the first step of the base excision repair (BER) pathway. NEIL1 recognizes and cleaves mainly oxidized pyrimidines from DNA. The past decade has witnessed the identification of an increasing number of post-translational modifications (PTMs) in BER enzymes including phosphorylation, acetylation, and sumoylation, which modulate enzyme function. In this work, we performed the first comprehensive analysis of phosphorylation sites in human NEIL1 expressed in human cells. Mass spectrometry (MS) analysis revealed phosphorylation at three serine residues: S207, S306, and a third novel site, S61. We expressed, purified, and characterized phosphomimetic (glutamate) and phosphoablating (alanine) mutants of the three phosphorylation sites in NEIL1 revealed by the MS analysis. All mutant enzymes were active and bound tightly to DNA, indicating that phosphorylation does not affect DNA binding and enzyme activity at these three serine sites. We also characterized phosphomimetic mutants of two other sites of phosphorylation, Y263 and S269, reported previously, and observed that mutation of Y263 to E yielded a completely inactive enzyme. Furthermore, based on sequence motifs and kinase prediction algorithms, we identified the c-Jun N-terminal kinase 1 (JNK1) as the kinase involved in the phosphorylation of NEIL1. JNK1, a member of the mitogen activated protein kinase (MAPK) family, was detected in NEIL1 immunoprecipitates, interacted with NEIL1 *in vitro*, and was able to phosphorylate the enzyme at residues S207, S306, and S61.

## Introduction

BER is initiated by a lesion-specific DNA glycosylase, which catalyzes the cleavage of the N-glycosidic bond, generating an apurinic/apyrimidinic (AP) site [1–3]. In humans, there are 5 DNA glycosylases specific for oxidized lesions that are grouped into two structurally distinct families: The helix-hairpin-helix (HhH) family comprises two members: a homolog of prokaryotic Nth, Nth-like 1 (NTHL1), and 8-oxoguanine (8-oxoG) DNA glycosylase (OGG1). Three endonuclease VIII (Nei) -like (NEIL) enzymes NEIL1, 2, and 3 belong to the (Fpg)/Nei family [4–10]. The NEIL enzymes possess two structurally conserved motifs, namely the helix-two-turns-helix motif and a zinc (or zincless in the case of NEIL1) finger motif, both of which are involved in binding to DNA [11]. NEIL1 primarily excises oxidized pyrimidines and hydantoin lesions from duplex DNA, and can cleave lesions in single-stranded, bubble, and bulge DNA substrates [6, 12, 13]. It has also been implicated in the removal of DNA lesions during nucleotide excision repair [14].

Post-translational modifications (PTMs) such as phosphorylation, methylation, alkylation, ubiquitylation, and sumoylation play critical roles in many aspects of DNA metabolism, cell-cycle regulation, and other cellular processes [15, 16]. PTMs provide a means to alter the function of respective protein targets thereby eliciting various responses such as determining sub-cellular localization, distinguishing protein binding partners, altering DNA binding activity, and modulating enzyme turnover [17, 18]. The integrity of DNA is challenged every day by numerous exogenous and endogenous factors [19]. The DNA damage response relies heavily on PTMs to sense the damage, trigger a response, and restore the cell to a damage-free state [20]. Reactive oxygen species (ROS) generated in the cell as a result of byproducts of respiration, ultraviolet light, and ionizing radiation lead to the generation of potentially lethal or mutagenic oxidative DNA damage [21, 22]. The highly coordinated base excision repair (BER) pathway is the major pathway involved in the repair of oxidized base lesions [23–27] and regulation of this pathway is further promoted by PTMs [17, 28, 29].

PTMs have been identified in all five human DNA glycosylases specific for oxidized lesions [17, 29–35]. Some of these sites were identified in high-throughput mass-spectrometry proteomic analyses and further characterized and evaluated using techniques such as site-specific mutagenesis, and *in vitro* activity assays [30, 32, 33]. For instance, the OGG1 DNA glycosylase is modified by c-Abl, and Cdk4 kinases [30]. A phosphorylation event by Cdk4 was reported to increase OGG1 incision activity whereas phosphorylation by c-Abl seemed to have little to no effect on the enzyme’s activity *in vitro* [30]. However, the precise sites of phosphorylation by these kinases still remain to be elucidated. Another study reported that acetylation of K338 and K341 in OGG1 modulated the enzyme’s repair activity in response to hydrogen peroxide treatment [36, 37]. Among the NEIL enzymes, the NEIL2 DNA glycosylase is reported to be acetylated at positions K49 and K153; while the latter has apparently no effect on enzyme activity, acetylation of residue K49 results in an inactive enzyme [32]. MUTYH, another mammalian DNA glycosylase that cleaves adenine opposite 8-oxoG was reported to be phosphorylated at S524. A phosphomimetic mutation of S524 (S524D) results in an enzyme with ~10-fold lower affinity for DNA [33].

**Fig 1:**
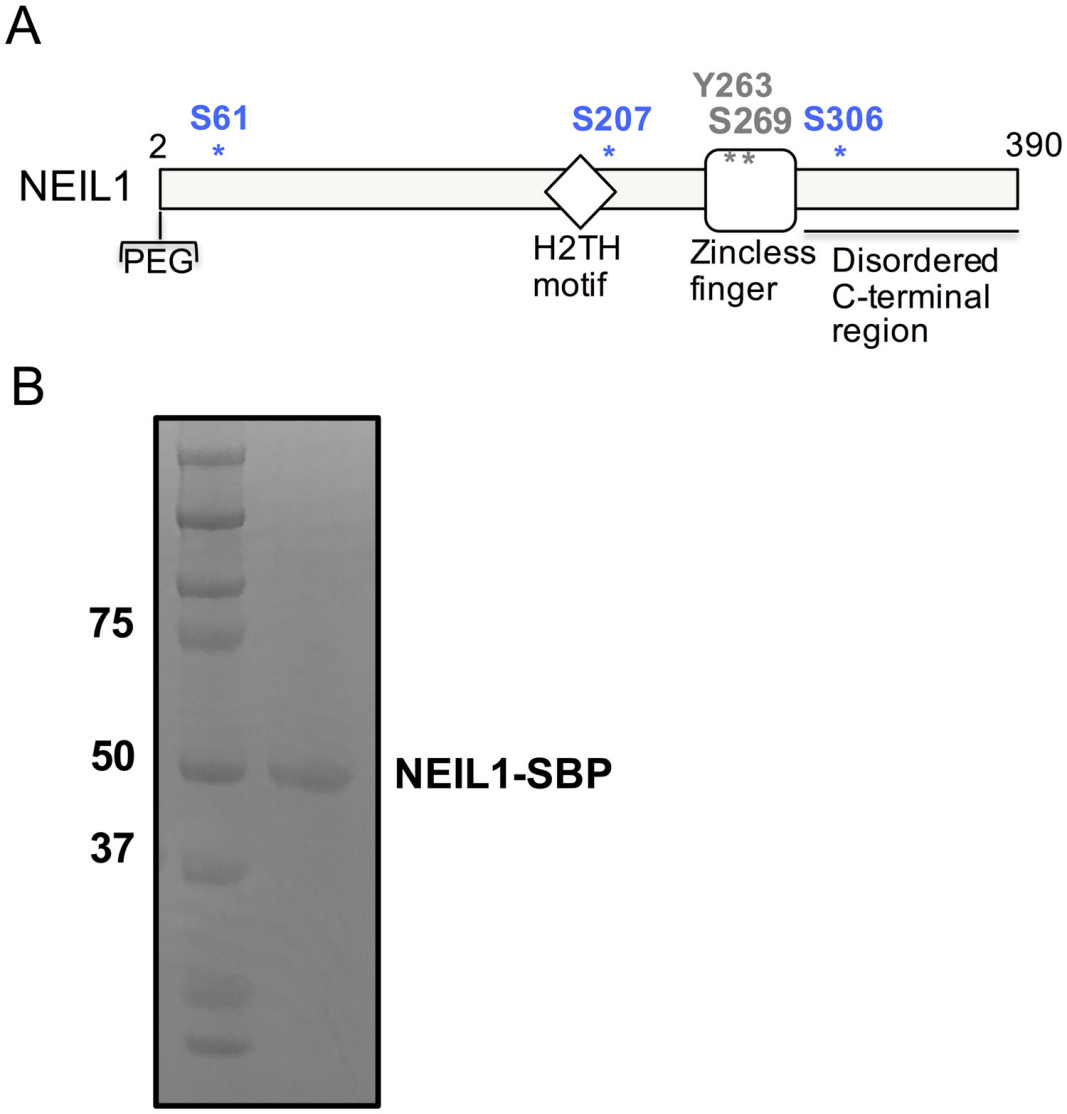
Sites of phosphorylation within the NEIL1 DNA glycosylase. (A) Domain map of NEIL1 indicating the position of known sites of phosphorylation. The residues S207, S306, and S61 identified in this study are shown in blue and the Y263 and S269 sites previously identified [39] are indicated in black. (B) SDS-PAGE gel of SBP-tagged NEIL1 after affinity pull-down from HEK293T cell-extracts overexpressing NEIL1. The gel was stained with Coomassie blue and the NEIL1-SBP band was cut from the gel and digested with trypsin for identification of phosphorylated peptides via LC-MS/MS.

There are currently four phosphorylation sites for NEIL1 reported in the literature: Y263, S269, S207, and S306 (Fig 1A) [38–40]. These sites were identified in high-throughput mass spectrometry screens but were not individually confirmed or characterized. We investigated the PTMs in the NEIL1 DNA glycosylase produced by expression of the enzyme in mammalian cells. We identified 2 of the 4 sites of phosphorylation observed previously, namely S207 and S306. In addition, we also identified a third, novel phosphorylation site, S61. Using a systematic approach we expressed, purified, and characterized phosphomimetic and phosphoablating mutants of the NEIL1 enzyme for the three serine sites identified by us using mass spectrometry, as well as the two other sites, Y263 and S269, identified elsewhere [39]. While most mimetic enzyme variants were active and possessed both glycosylase and lyase enzyme activities the Y263E mutation rendered the enzyme inactive. In addition, we identified the c-Jun N-terminal kinase 1 (JNK1) as the kinase involved in the phosphorylation of NEIL1 at S207, S306, and S61.

## Materials and Methods

### Cloning, Overexpression, and Purification of NEIL1

The full-length human NEIL1 construct was cloned and purified as described previously [41]. The protein was expressed in Rosetta2 (DE3) pLysS *Escherichia coli* cells (Novagen), followed by IPTG induction (0.6 mM) for 4 hrs at 25°C. The cell pellet was resuspended in a buffer containing 50 mM sodium phosphate, pH 8.0, 150 mM NaCl, 10 mM imidazole pH 8.0, 10% (v/v) glycerol, 5 mM β-Me, and 1 mM PMSF and sonicated. The clarified cell lysate was added to pre-equilibrated TALON cobalt resin (Clontech). The proteins were eluted using 250 mM imidazole in the above buffer and diluted two fold into a buffer containing 20 mM Tris-HCl, pH 7.5, 300 mM NaCl, 10% (v/v) glycerol, and 1 mM DTT. A HiTrap SP-FF column (GE healthcare) with a linear NaCl gradient (300 mM– 1 M) was used to elute the enzymes and the resulting fractions were exchanged into a buffer containing 20 mM Tris-HCl, pH 7.5, 300 mM NaCl, 10% (v/v) glycerol, and 1 mM DTT. A Superdex 200 gel-filtration column (GE Healthcare) was used as a final purification step after which the enzyme was concentrated and flash frozen in LN_2_ for long term storage at -80°C. A QuikChange XL II site-directed mutagenesis kit (Stratagene) was used to introduce the S207E, S207A, S306E, S306A, S61E, S61A, S207E/S306E, S207A/S306A, S207E/S306E/S61E, and S207A/S306A/S61A mutations in the NEIL1 plasmid. All mutant enzymes were expressed and purified in a manner similar to the WT enzyme (incubation temperature: 25°C, 0.6 mM IPTG, cells harvested after 4 hours) with these exceptions: Double and triple mutants (DE, DA, TE, TA): 25°C, 0.4 mM IPTG, 3 hours; Y263E/Y263F: 16°C, 0.4 mM IPTG, overnight.

### Cell culture, NEIL1 expression, and pull-down of NEIL1 from nuclear extracts

HEK293T cells were cultured in DMEM containing 10% FBS and 1% Penicillin/Streptomycin at 37°C with 5% CO_2_. A pCC384 vector containing NEIL1 with a C-terminal streptavidin binding protein (SBP) tag (kind gift from Dr. David Pederson, University of Vermont) was transiently transfected into the HEK293T cells using Turbofect (Thermo Fisher) as detailed in the manufacturer’s protocol. The cells were supplemented with fresh media 16 hrs post transfection and were washed and scraped into cold PBS 48 hrs later. Nuclear extracts were prepared as described previously [42, 43]. Briefly, cells were lysed in a hypotonic lysis buffer (HLB) buffer containing 25 mM HEPES, pH 8, 1 mM EGTA, 2 mM magnesium chloride, 1 mM DTT, 3 mM sodium butyrate, 1 mM sodium pyrophosphate, 1 mM B-phosphoglycerate, 1 mM sodium vanadate, and 1 protease inhibitor tablet (Roche). Cells were lysed using 20 strokes on a Dounce homogenizer. Nuclei were pelleted by centrifugation at 8000 x g. The nuclei were resuspended in buffer HLB supplemented with 500 mM NaCl and were allowed to sit on ice for 20 min followed by lysis using 20 strokes on a Dounce homogenizer. The nuclear extract was obtained after centrifugation at 13,000 x g for 15 min at 4°C. Streptavidin magnetic C1 beads (Thermo Fisher Scientific) were equilibrated in HLB and incubated with the nuclear extract with gentle agitation for 2 hrs. A magnetic rack was used to collect the beads that were subsequently washed 3 times to remove non-specifically bound protein. The NEIL1-SBP protein was boiled off from the beads in Laemmli buffer and was run on an SDS-PAGE gel, and stained with Coomassie blue. For immunoprecipitation (IP) reactions, a NEIL1-FLAG vector was transfected into HEK293T cells and the IP was performed with an anti-FLAG M2 antibody (Agilent 200470–21, Lot# 0006057602) as described previously [44].

### Mass Spectrometry Analysis

Individual bands containing NEIL1-SBP were excised from the SDS-PAGE gel and were prepared for MS analysis using standard tryptic digest procedures [45]. Gel lanes were cut into 1-mm^3^ pieces and destained completely with 50 mM ammonium bicarbonate in 50% acetonitrile. Protein samples were reduced in 10 mM DTT at 55°C for 1 hr and alkylated with 55 mM iodoacetamide. The gel pieces were washed, rehydrated and dehydrated twice alternating between 100 mM ammonium bicarbonate and 100% acetonitrile and then dried in a SpeedVac (Scientific Support, Hayward, CA, USA). Trypsin digestion was carried out for 18 hours at 37°C with 7 ng/μl of trypsin. The tryptic peptides were acidified with 5% formic acid to stop the reaction, extracted, dried in a SpeedVac and stored at -80°C until LC-MS analysis. The dried peptide samples were re-suspended in a solution of 2.5% acetonitrile and 2.5% formic acid in water.

LC-MS based protein identification was performed on a linear ion trap (LTQ)-Orbitrap Discovery mass spectrometer coupled to a Surveyor MS Pump Plus (Thermo Fisher Scientific, Waltham, MA, USA). Five μL was loaded onto a 100 um x 120 mm capillary column packed with MAGIC C18 (5 μm particle size, 20 nm pore size, Michrom Bioresources, CA) at a flow rate of 500 nl/min. Peptides were separated by a gradient of 5–35% acetonitrile and 0.1% formic acid for 98 min, 40–100% acetonitrile and 0.1% formic acid for 1 minute, and 100% acetonitrile for 10 min. Separated peptides were introduced into the linear ion trap via a nanospray ionization source and a laser pulled ~3 μm orifice with a spray voltage of 1.8 kV. An Orbitrap survey scan from *m/z* 360–1600 at 30,000 (FWHM) resolution was paralleled by 10 collision-induced dissociation MS/MS scans of the most abundant ions in the LTQ. MS/MS scans were acquired with the following parameters: isolation width: 2 *m/z*, normalized collision energy: 35%, activation Q: 0.250, and activation time = 30 ms. Review mode for FTMS master scans was enabled. Dynamic exclusion was enabled (repeat count: 2; repeat duration: 30 sec; exclusion list size: 180; exclusion duration: 60 sec). Singly charged ions were excluded for MS/MS.

Product ion spectra were searched using the SEQUEST search engine on Proteome Discoverer 1.4 (Thermo Fisher Scientific, MA) against a curated Human database with sequences in forward and reverse orientations. Search parameters allowed for full trypsin enzymatic activity, two missed cleavages, and peptides between the MW of 350–5000 Da. Search parameters set the mass tolerance at 20 ppm for precursor ions and 0.8 Da for fragment ions, dynamic modifications on methionine (+15.9949 Da: oxidation), serine, threonine, and tyrosine (+79.9663 Da: phosphorylation) with a maximum of 4 dynamic modifications allowed per peptide; and static modification on cysteine (+57.0215 Da: carbamidomethylation). The Scaffold software 4.3 (Proteome Software, OR) was then used to further analyze the results. Cross-correlation (Xcorr) significance filters were applied in the initial analysis where Xcorr values were >1.5 for ions that were singly charged (+1), >2.0 for ions that were doubly charged (+2), and >2.5 for ions that were triply charged (+3). Other filters applied were a minimum peptide cutoff of 2 as well as DeltaCN >0.1. MS/MS spectra of the phosphorylated peptides were manually evaluated. All spectra are displayed as figures in the manuscript as well as in the supplemental information and are therefore not deposited in a public repository.

### DNA Substrates

The 35-mer oligodeoxynucleotides used for the glycosylase/lyase activity assays were purchased from Midland Certified Reagent Co. (Midland, TX) and purified by urea PAGE. The sequence of the damage-containing strand was 5′-TGTCAATAGCAAG(X)GGAGAAGTCAATCGTGAGTCT-3′, where X was Tg, 5-OHU, DHU, or uracil, which was used to create an apurinic/apyrimidic site (AP-site). The complementary oligonucleotide had the following sequence: 5′-AGACTCACGATTGACTTCTCC(C)CTTGCTATTGACA-3′. Spiroiminodihydantoin (Sp) was synthesized as described previously [46] in the following sequence context: 5′-TGTTCATCATGCGTC(Sp)TCGGTATATCCCAT-3′. The complementary strand for Sp DNA was 5′-ATGGGATATACCGA(C)GACGCATGATGAACA-3′. End-labeling of substrates was carried out on 1 pmole of each damage-containing strand using T4 polynucleotide kinase (New England Biolabs, Ipswich, MA) in presence of [γ-^32^P] dATP, for 30 min at 37°C. The phosphorylation reaction was terminated by addition of 25 mM EDTA and heat inactivation. The end-labeled DNA was separated from the [γ-^32^P] dATP by ethanol precipitation and diluted in 9 pmoles of the appropriate non-labeled damage-containing oligodeoxynucleotide and 10 pmoles of the complementary oligodeoxynucleotide to a final concentration of 250 nM in 10 mM Tris—HCl (pH 8.0) and 50 mM NaCl. In order to create an AP site, a double-stranded uracil-containing oligodeoxynucleotide was treated with 2 units of uracil DNA glycosylase (New England Biolabs, Ipswich, MA) for 30 min at 37°C.

### Glycosylase/Lyase Activity Assays

Glycosylase/lyase assays were performed with 20 nM lesion-containing substrate and increasing concentrations of enzyme in 20 mM HEPES pH 7.5, 150 mM NaCl, 2 mM EDTA with 200 μg/ml BSA at 25°C for 30 mins. The reactions were stopped with the addition of an equal volume of formamide loading buffer (98% formamide, 5 mM EDTA, 0.1% xylene cyanol and 0.1% bromophenol blue) to assay for both glycosylase and lyase activities. The reaction products were separated from the uncleaved substrates with a 12% (w/v) denaturing polyacrylamide gel and quantified with an isotope imaging system (Molecular Imaging System, Bio-Rad).

### Electromobility shift assays

Gel shift analysis was performed using 35-mer double stranded oligos with tetrahydrofuran in the lesion position using the same sequence context as above. The furan-containing strand (1 pmol/μl) was labeled with T4 PNK in presence of [γ-^32^P] dATP, for 30 min at 37°C. The same amount of complimentary DNA was added to the mixture and allowed to anneal to the labeled damaged strand thereby yielding 100% labeled duplex. Samples (without loading dye) containing 10 pM DNA and varying amounts of enzyme (0–600 nM) were loaded onto a pre-run 10% native polyacrylamide gel. 2x loading dye was loaded in one lane as a marker. The gels were run for 2 hrs at 10 mA and then transferred to Whatmann 3 mm paper, covered with plastic wrap and placed on a gel drier at 80°C for 1 hr. The dried gels were exposed to a K-screen overnight and imaged with a Molecular Imaging System (BioRad).

### Western Blotting and far-Western Analysis

Samples for far-Western analysis were boiled in SDS-sample buffer and loaded on a 4–12% precast gradient gel (Novex) and run for 1 hr at a constant 180 V. The gels were transferred to an Immobilon^®^-FL PVDF membrane (Millipore). The membrane was washed (2X for 10 min each) with 1X PBS and 1 mM DTT. The membrane was then incubated in PBS with 6M guanidine-HCl and 1 mM DTT for 30 min at room-temperature with gentle shaking. The proteins on the membrane were then gradually refolded using serial dilutions of guanidine-HCl (in PBS with 1 mM DTT) to a final concentration of 0.09M guanidine-HCL (6M, 3M, 1.5M, 0.75M, 0.375M, 0.1875M, and 0.09M). The first 5 steps were done at room temperature for 30 minutes. The penultimate and final steps were performed overnight at 4°C. The membranes were then blocked for 1 hr using PBS blocking reagent (LI-COR Biosciences) and further incubated with a pre-prepared HEK293T cell lysate (1 mg/ml in Cell Lytic M buffer, Sigma) overnight. The membranes were then washed (3X) with cold PBS containing 0.05% Tween-20 and then processed using a standard Western blot protocol (LI-COR Biosciences). An anti-mouse JNK1 purified monoclonal antibody (for far-Western analysis, clone 228601 from R&D Systems, lot # UQL0209081; cat # MAB17761) and a rabbit polyclonal NEIL1 antibody (for Western Blotting of IP reactions, Abcam, ab21337, lot # GR133490-1) were diluted 1:1000 (final concentration 1μg/ml) in blocking reagent. The blots were then incubated with anti-mouse/rabbit secondary antibody (LI-COR Biosciences, IR Dye 800-CW, Goat-anti-mouse: cat # 926–32210, lot #: C40213-01 and IR Dye 680, Goat-anti-rabbit: catalog # 926–68071, lot #: C31205-05) and scanned using an Odyssey CLx imaging system (LI-COR biosciences).

### *In vitro* Kinase Assay

NEIL1-WT and phosphomimetic or ablating mutants (10 μM) were incubated in 1X kinase buffer (Cell Signaling), supplemented with 2 mM ATP (Cell Signaling), 0.2 μl γ-^32^P ATP (Perkin Elmer) and 25 ng/μl purified active mouse JNK1 (Abcam ab60304, Serial: X5612-3894). Reactions (20 μl total volume) were incubated at 32°C for 30 minutes. The reaction was stopped by the addition of 5 μl 6X SDS-PAGE sample buffer and boiled at 95°C for 5 minutes. Samples were resolved by SDS-PAGE, the gel was dried, and phosphorylation via γ-^32^P incorporation was visualized using autoradiography. MS analysis was used to verify sites of phosphorylation. Samples for MS were incubated without γ-^32^P ATP and stained using Coomassie Blue. Bands were excised and digested with trypsin followed by MS/MS analysis performed as described above.

## Results

### Identification and location of phosphorylation sites in NEIL1

**Fig 2:**
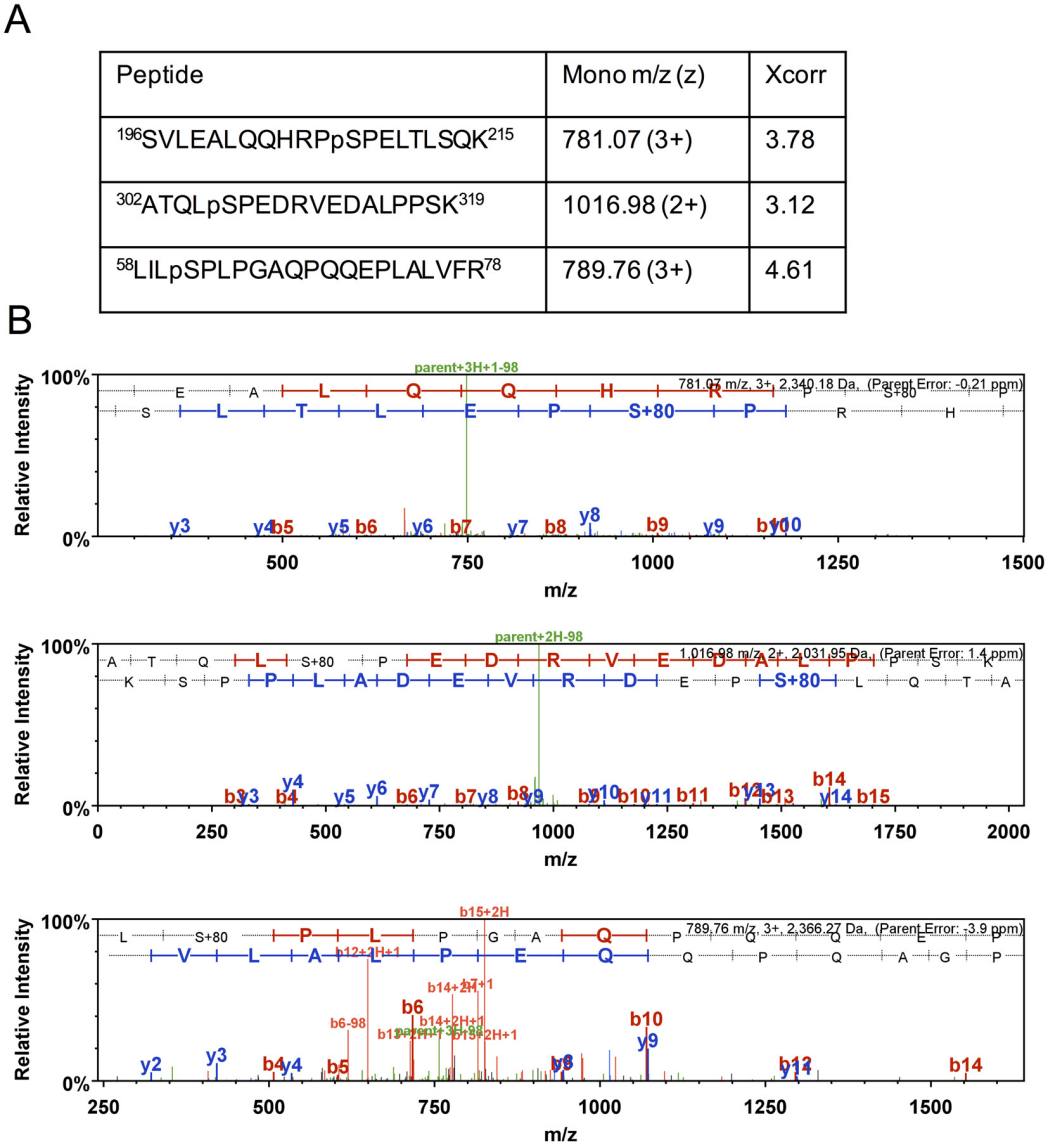
Identification of phosphorylation sites on NEIL1 by LC-MS/MS. (A) List of phosphorylated peptides identified from LC-MS/MS analysis of NEIL1-SBP with their corresponding monoisotopic mass m/z, charge-state (z), and Xcorr values. (B) Product-ion spectra (Scaffold software 4.3) for each phosphorylated peptide are displayed. The b- and y-ions are shown in red and blue, respectively, and the neutral loss peak resulting from the loss of a phosphogroup from the parent ion is indicated in green.

In order to identify PTMs in the NEIL1 DNA glycosylase, a construct of NEIL1 tagged at the C-terminus with streptavidin binding peptide (SBP) was transfected into HEK293T cells. NEIL1-SBP was isolated using streptavidin magnetic beads, run on an SDS-PAGE gel, and stained with Coomassie blue (Fig 1B). The single protein band was cut from the gel, digested with trypsin and the resulting peptides were subjected to mass spectrometry (MS) analysis. NEIL1 was identified as the dominant species in the sample (>50% peptide coverage) and four sites of phosphorylation were identified including S207, S306, S61, and S344 (Fig 2). The latter was seen only once and was not further evaluated herein, whereas the other three sites were observed at least twice and as many as four times in our analyses from separate experiments. Spectra obtained for each site were manually evaluated after an initial search using the SEQUEST search engine on Proteome Discoverer 1.4. Peptides containing the three phosphorylation sites are reported in Fig 2A with their corresponding monoisotopic mass:charge (m/z) values, peptide charge states (z), and Xcorr values. The annotated MS/MS spectra are shown in Fig 2B. Accurate mass measurements of these phosphopeptides demonstrated that all peptide masses measured were within 10 ppm of the theoretical masses (with an error of -0.21 ppm, 1.4 ppm, and -3.9 ppm for S207, S306, and S61, respectively). The tandem mass spectra exhibit a continuous stretch of b- and y-ion series, with high Xcorr scores. MS analysis of the phosphopeptides by collision-induced dissociation are often accompanied by a loss of a phosphogroup from the parent ion (neutral loss), which was observed in the spectra of the phosphopeptides containing S207, S306, and S61 (indicated in green, Fig 2B). Y263 and S269, the two sites previously reported to be phosphorylated in a high-throughput mass-spectrometry study, were not identified in our preparations [39]. Given that the relative abundance of phosphotyrosine is much lower (1.8%) than that of phosphoserine (86.4%) and phosphothreonine (11.8%), it is not surprising that we were unable to detect the previously reported phosphorylated Y263 [47].

Based on the MS data obtained, we focused on the three serine sites, S207, S306, and S61. We generated phosphomimetic (glutamate) and phosphoablating (alanine) variants at each of the three serine positions in NEIL1 [48]. (Table 1).

**Table 1:** Phosphomimetic/ phosphoablating mutants of NEIL1.

NEIL1 PTMs	Literature	Identified in this work by MS analysis (seen >1)	Phosphomimetic/ phosphoablating mutants	Expression conditions*	Glycosylase/ lyase activity	Kd (nM)
			WT	25°C, 4 hrs, 0.6 mM IPTG	+	23 ± 3.9
S207	[40]	Yes				
	This work		S207E	*	+	4.5 ± 0.8
			S207A	*	+	3.2 ± 1.1
S306	[38]	Yes				
	This work		S306E	*	+	7.8 ± 2.2
			S306A	*	+	2.3 ± 0.3
S61	This work	Yes				
			S61E	*	+	19.6 ± 3.5
			S61A	*	+	16.2 ± 3.8
Double Mutants	This work		S207E/S306E (DE)	*	+	15 ± 2.1
			S207A/S306A (DA)	*	+	10.8 ± 1.3
Triple Mutants	This work		S207E/S306E/S61E (TE)	25°C, 3 hrs, 0.4 mM IPTG	+	1.3 ± 0.27
			S207A/S306A/S61A (TA)	*	+	13.2 ± 3
Y263	[39]	No				
			Y263E	16°C, o/n, 0.4 mM IPTG	-	n/a
			Y263F	*	+	n/a
S269	[39]	No				
			S269E	*	+	n/a
			S269A	*	+	n/a

* Conditions are the same as the WT enzyme.

In addition to NEIL1 harboring single point mutations, we created double S207E/S306E (DE) and triple S207E/S306E/S61E (TE) mutations, as well as the corresponding double and triple Ala mutations (DA and TA, Table 1). The S207 and S306 residues are present in disordered regions in the crystal structure and based on sequence alignments of mammalian Neil1 enzymes, S207 is well-conserved through species, while S306 appears to be less conserved (S1A and S1B Fig). S61 is highly conserved through species and is present at the tip of β-strand 3 located in the ordered N-terminal domain of the enzyme (S1A and S1B Fig). Despite the fact that we were unable to identify phosphorylated peptides for Y263 and S269 in our mass spectrometry data, we engineered the mimetic and ablating mutations for these residues, both of which are located in the highly conserved, zincless-finger domain of the enzyme (S1A and S1B Fig).

### Expression, Activity, DNA binding, and turnover of NEIL1 phosphomimetic and phosphoablating mutants

NEIL1 carrying each of the phosphomimetic/ phosphoablating mutations was expressed and purified in a similar manner as the WT enzyme. A three-step purification scheme using TALON metal affinity chromatography, cation exchange, and size exclusion was employed for the purification of all NEIL1 constructs. While most of the mutants behaved much like the WT enzyme during the expression and purification steps, it took multiple attempts and different expression conditions to express and purify the enzymes harboring the TE and the Y263E mutations (see Table 1 for differences). Despite numerous efforts to purify the TE enzyme to near homogeneity, several contaminants were still observed on an SDS-PAGE gel after the gel-filtration step (S1C Fig).

**Fig 3:**
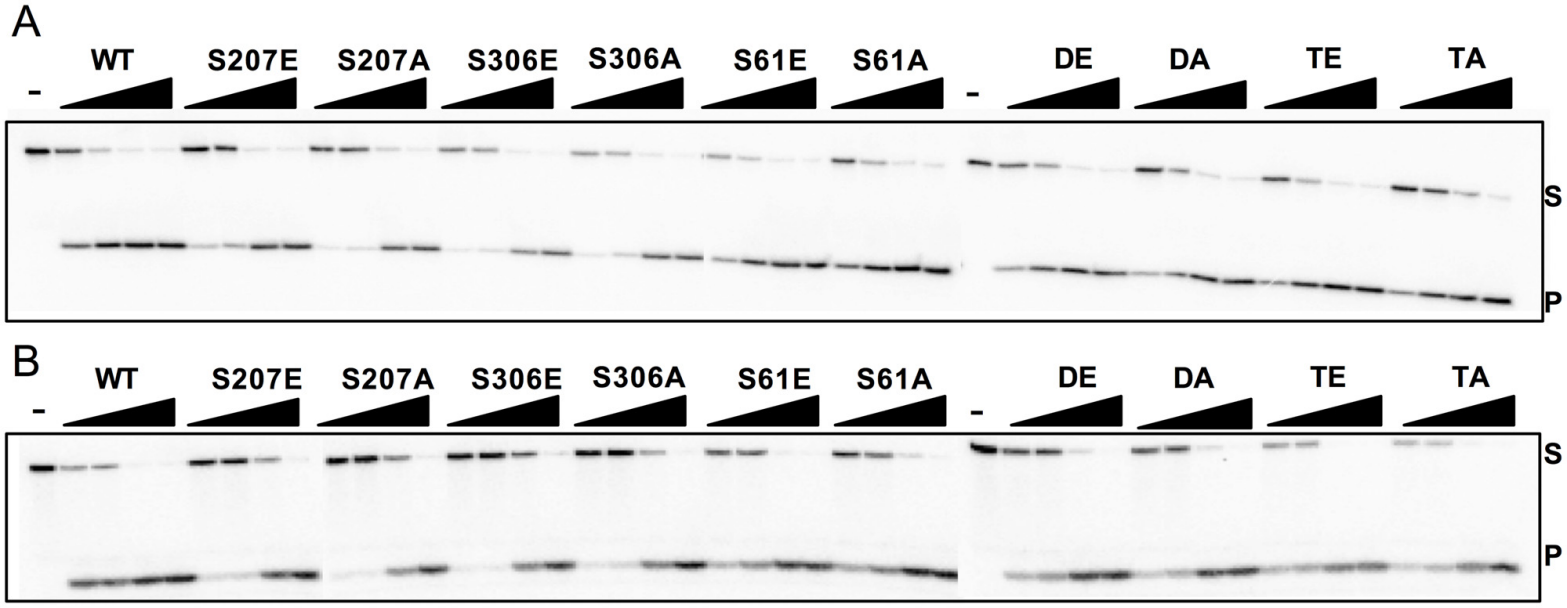
Glycosylase and lyase activity panel for human NEIL1-WT and the phosphomimetic/ablating mutants. Glycosylase assays were performed by incubating 20 nM of double-stranded DNA substrates (A) Sp:C and (B) AP:C and increasing amounts of enzyme with the following substrate to enzyme ratios: 1:0.5, 1:1, 1:4, and 1:16. “-” indicates a no enzyme negative control. Assays were performed at room temperature for 30 minutes. S and P indicate substrate and product, respectively. Data shown are representative of duplicate experiments.

We tested the glycosylase and lyase activities of WT NEIL1 and the variants using oligonucleotides containing Sp placed opposite C (Sp:C, Fig 3A) and an abasic (AP) site opposite C (AP:C, Fig 3B). All proteins containing mutations of S207, S306, and S61 possessed both glycosylase and lyase activities similar to WT indicating that these enzymes are well-folded and that mutation to either Glu or Ala does not affect the enzyme’s catalytic function under the conditions employed in this assay (Fig 3). The Y263E mutation yielded a completely inactive enzyme (S2 Fig), a result that is not surprising given that this residue would likely make direct H-bond contacts with the DNA backbone based on the structural superposition of NEIL1 with its viral ortholog, MvNei1 bound to DNA ([49] S1B Fig). The negative charge imparted by the glutamate residue in place of the tyrosine would likely result in unfavorable interactions with the DNA. The Y263F and S269E/A mutants did not display the impaired activity that was observed with the Y263E mutation (S2 Fig).

**Fig 4:**
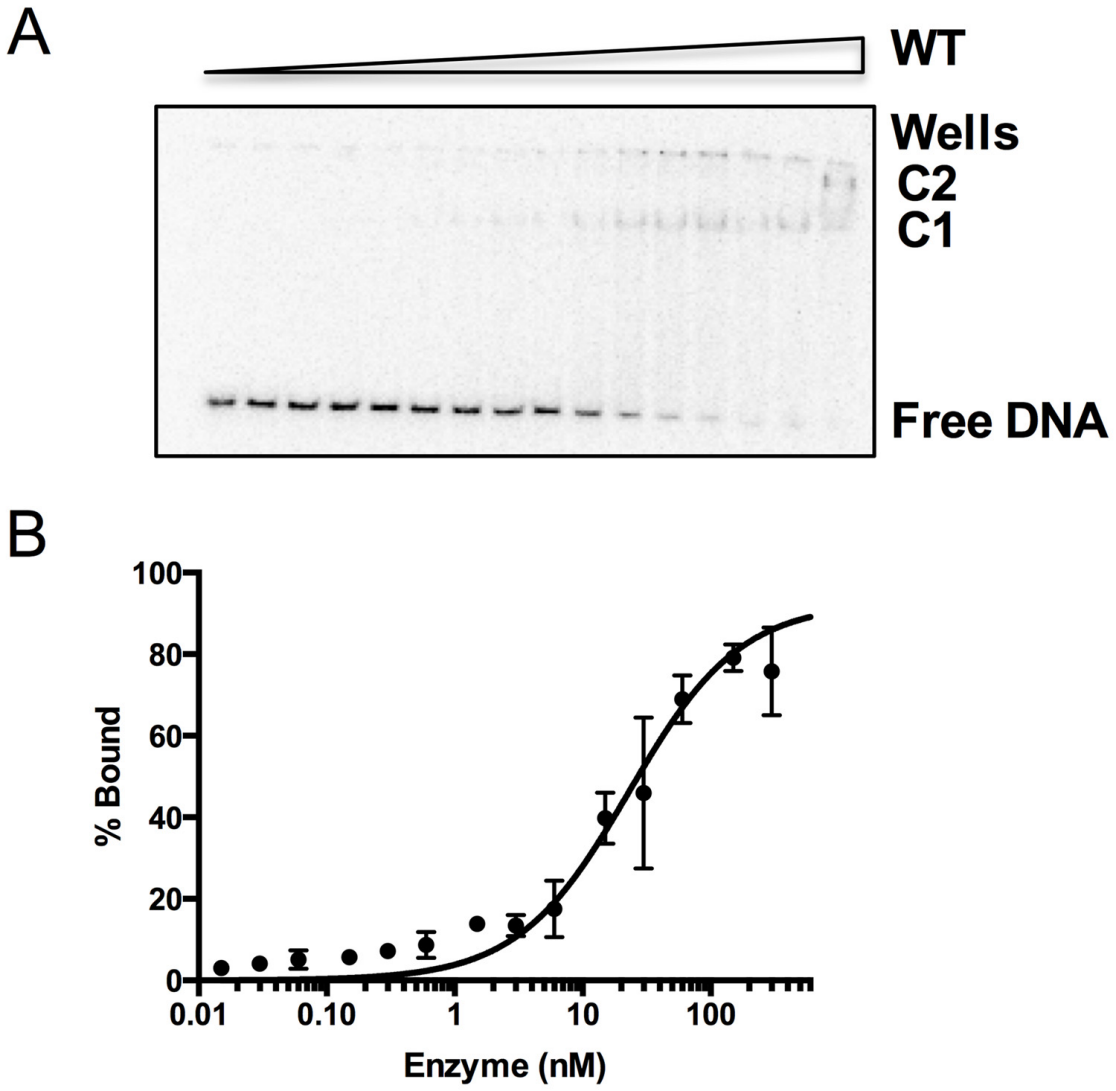
Binding of NEIL1 and mutant enzymes to DNA. (A) Representative phosphor-autodiogram for binding of NEIL1-WT to a 35-mer furan-containing DNA where the substrate (10 pM) was incubated with increasing (0–600 nM) amounts of enzyme. Complex formation is indicated by the presence of shifted bands C1 and C2. (B) Graphical fitting of the EMSA data indicated above using GraphPad Prism 6. The data were fit to the one-site specific binding equation. The K_d_ values for WT and the NEIL1 mutants are listed in Table 1 and are representative of experiments performed in duplicate.

In order to assess the affinity of the variants for DNA, electrophoretic mobility shift assays (EMSAs) were performed using an uncleavable abasic site analog, tetrahydrofuran, substrate paired opposite C with increasing amounts of either NEIL1-WT or a phosphomimetic/ phosphoablating mutant. Free DNA and shifted bands representing complex (C) formation were quantified. A representative EMSA reaction for the WT enzyme is shown (Fig 4A) accompanied by a graphical plot where the percentage of protein bound is graphed versus enzyme concentration (Fig 4B). A K_d_ value was extrapolated for each mutant using the one-site specific binding equation (Prism 6.0, Table 1). All mutant enzymes possessed WT or slightly better than WT affinity for DNA with K_d_ values ranging from ~2–20 nM (Table 1, S3B Fig). Higher order complex formation occurred only at the highest enzyme concentration for NEIL1-WT, but other mutants such as S207A displayed formation of more than one complex even at lower enzyme concentrations (S3A Fig). In such cases, all shifted bands representing a complex (C1+C2+C3) were quantified and graphed (S3B Fig). The upper bands (C2 and C3) could be a result of non-specific complex formation as seen with the either the Fpg or MutY enzymes reported previously [50] and may contribute to the lower K_d_ values observed for S306A, S207A, and the TE variants.

Since mutants of S207, 306, and 61, possessed both glycosylase and lyase activities under the conditions tested above (Fig 3), we used multiple turnover assays under conditions where [enzyme]<[substrate] to test the effects of mimicking phosphorylation on enzyme turnover. Under the conditions chosen here, WT-NEIL1 displays biphasic behavior with an initial rapid “burst” phase followed by a slower steady-state phase (S4 Fig). While most of the enzymes harboring glutamate mutations displayed near WT bursts, the S306E and S61E mutants displayed a reduced ability to turnover (S4A Fig). This defect in the enzyme’s ability to turnover could be attributed to impaired product release relative to the location of the mutation. All the alanine mutants displayed a lower ability to turnover compared with the WT enzyme (S4B Fig). This mitigated ability for enzyme turnover displayed by the alanine variants could be associated with enzyme stability where mutation to alanine, a non-polar substitution not amenable to H-bond interactions, is much less tolerated than a mutation to glutamate. Along the same lines, stability assays conducted with a phosphoablating mutant in MUTYH, S524A, indicated that the alanine mutant was much less stable than the phosphomimetic mutant (S524E) counterpart [33]. Based on these data and the EMSA data above, it is likely that the alanine mutants S207A, S306A, and TA also interact non-specifically with the DNA, which also may explain their reduced ability to turnover. Binding of NEIL1 non-specifically to undamaged DNA has been documented elsewhere [51].

### The JNK1 protein kinase phosphorylates NEIL1 at S207, S306, and S61

The mitogen-activated protein kinase (MAPK) family of kinases phosphorylate Ser or Thr residues in response to extracellular stimuli and environmental stresses [52]. These kinases have an absolute requirement for a Pro residue at the +1 position following Ser or Thr in order for a phosphorylation event to occur. Sequence analysis of NEIL1 indicates that the enzyme has 3 Ser residues S207, S306, and S61 that are immediately followed by Pro (S5 Fig) and are therefore potential candidates for phosphorylation by members of the MAPK family. In order to narrow the pool of MAPKs that could potentially be involved in phosphorylating NEIL1 at these 3 sites, we used the webserver PhosphoNET (www.phosphonet.ca) [53]. For all three sites, the MAPK8 (or JNK1) kinase was the top predicted kinase followed closely by MAPK10 (JNK3), and ERK1/2. The mammalian JNK family comprises three kinases JNK1, JNK2, and JNK3, which play a role in cellular processes such as apoptosis, response to oxidative stress, and DNA damage signaling [52, 54].

**Fig 5:**
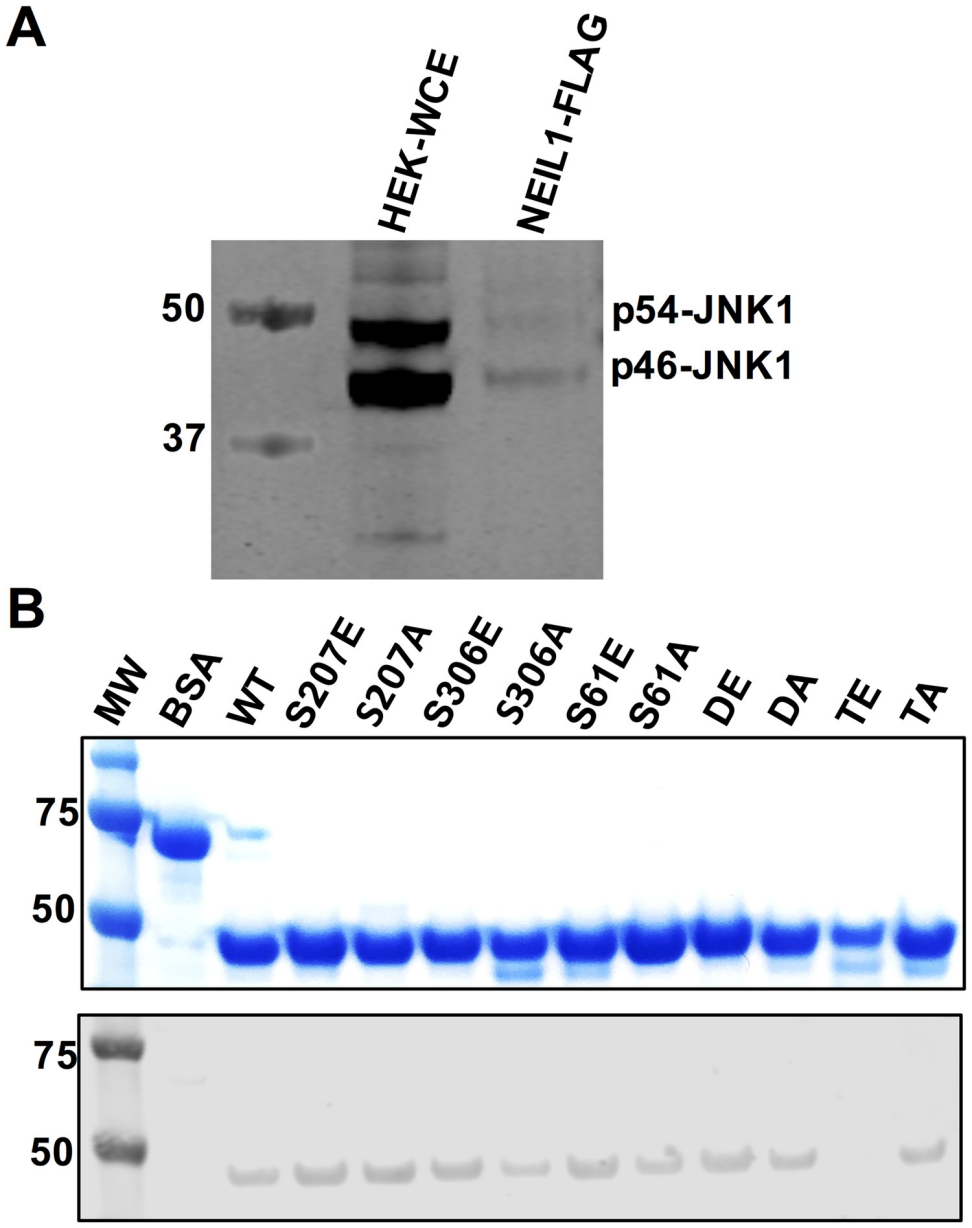
The JNK1 kinase interacts with NEIL1. (A) Western blot analysis of endogenous JNK1 present in HEK293T WCE and in NEIL1-FLAG immunoprecipitates. (B) Far-Western analysis to confirm the interaction between NEIL1 and JNK1. Top panel, Coomassie stained SDS-PAGE gel of NEIL1 and its phosphomimetic and ablating mutants (50 pmoles). Bottom panel, membrane after transfer, denaturation, slow refolding, binding to JNK1 from HEK293 WCE, and probing with an anti-JNK1 antibody. BSA was used as a negative control.

In order to test whether JNK1 and NEIL1 are able to interact in cells, we performed immunoprecipitation (IP) on HEK293T cells overexpressing a NEIL1-FLAG construct. Western blot analysis revealed that both isoforms of JNK1, p46 and p54, are present in HEK293T whole cell extracts (WCE), and are detected in the NEIL1-IP (Fig 5A). Using far-Western analysis, we tested whether NEIL1 is able to interact directly with JNK1 *in vitro*. NEIL1 and the phosphomimetic and ablating mutants were transferred and immobilized on a membrane after SDS-PAGE analysis, denatured with guanidine HCl, gradually refolded, and incubated with HEK293 WCE. Equal loading (50 pmol) of all mutants was attempted as indicated by the Coomassie stained gel (Fig 5B, top panel) and BSA was used as a negative control. We could not estimate an accurate protein concentration for the TE variant, as it was the only mutant that co-purified with several contaminating proteins. Far Western analysis confirmed that all mutants were able to bind JNK1 with the exception of the TE variant (Fig 5B, bottom panel). This result could be attributed to the lower amount of TE enzyme loaded on the gel as well as a potentially unstable interaction when all three serine residues are mutated to glutamate.

**Fig 6:**
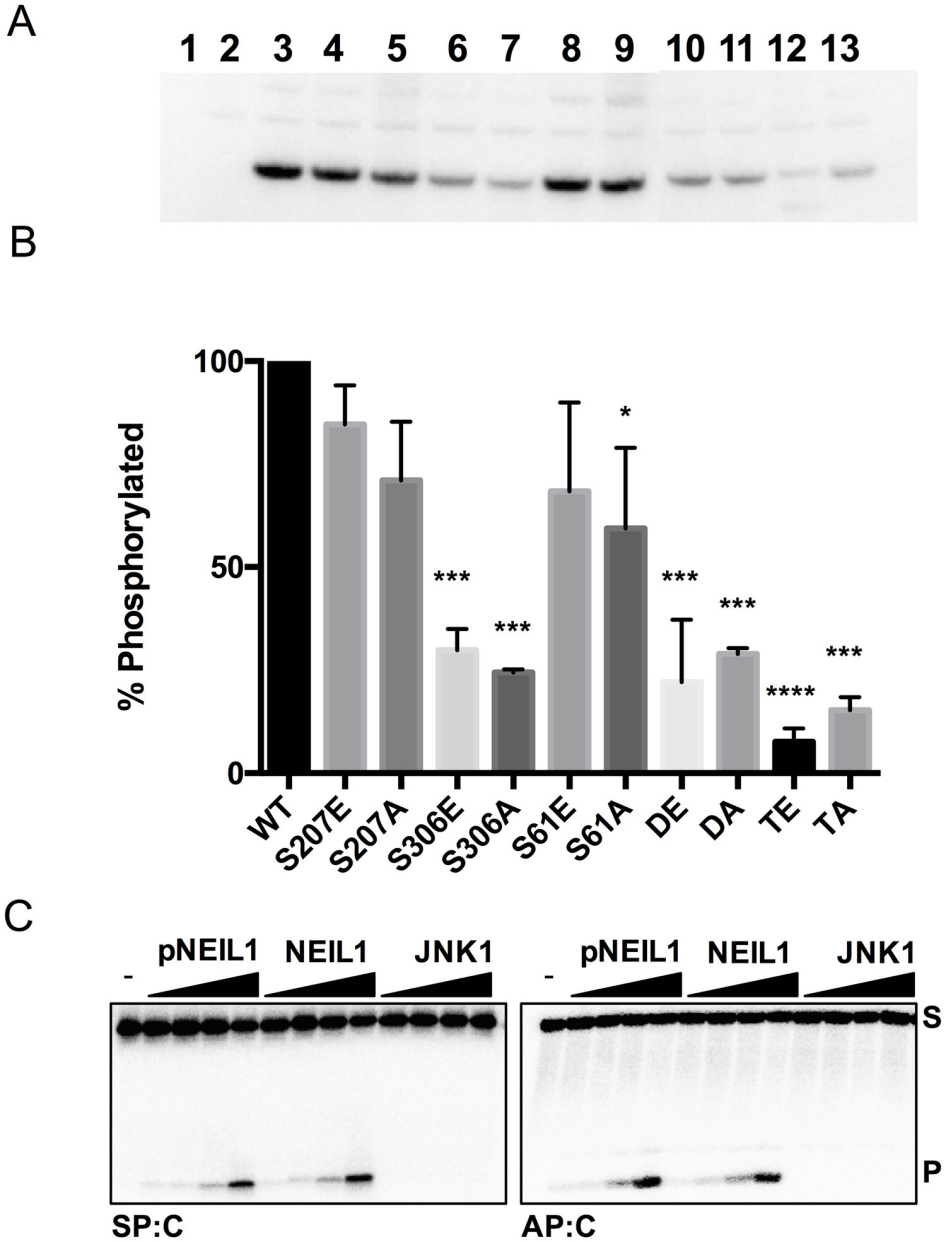
Phosphorylation of NEIL1 by JNK1 using an *in vitro* kinase assay. (A) *In vitro* kinase assays were performed with NEIL1 constructs expressed from E. coli cells and purified to homogeneity and active JNK1 kinase for 30 minutes at 32°C. γ-^32^P incorporation was quantified using phosphor-autoradiography after SDS-PAGE analysis of the samples. Lane 1, no JNK1 control; lane 2, no NEIL1 control; lane 3–13 are WT, S207E, S207A, S306E, S306A, S61E, S61A, DE, DA, TE, TA, respectively. (B) Graphical representation of two experimental repeats of the *in vitro* kinase assay. Statistically significant values (at 95% confidence) were determined by a one-way Anova test where * denotes p-values <0.05, *** denotes p-values <0.0005, and **** denotes p values <0.0001. (C) Glycosylase activity assays were performed using Sp:C and AP:C substrates with increasing amounts of *in vitro* phosphorylated NEIL1 (pNEIL1), unphosphorylated NEIL1 (positive control), and JNK1 (negative control). S and P indicate substrate and product, respectively.

In addition, we tested whether JNK1 could phosphorylate NEIL1 *in vitro*. Purified NEIL1 was treated with active JNK1 kinase in the presence of γ-^32^P ATP. SDS-PAGE analysis of the samples indicates that NEIL1-WT was phosphorylated *in vitro* (Fig 6A, lane 3). Only a modest decrease in ^32^P incorporation was observed when S207 and S61 were mutated to E or A (p values < 0.05 for the S61A mutant, Fig 6A lanes 2–5 and 8–9, Fig 6B), whereas, a statistically significant decrease in phosphor signal was observed for the S306E/A mutants and the double DE and DA mutants (with p values < 0.0005, Fig 6A, lanes 6–7 and 10–11, Fig 6B). The triple mutants, TE and TA displayed an even greater statistically significant decrease in percentage of phosphorylated protein with p values < 0.0001 (Fig 6A, lanes 12–13, Fig 6B). Since the most striking effects were observed by mutating S306 to E and A, this site is most likely the primary target for JNK1 phosphorylation. While S207 and S61 can be phosphorylated by the JNK1 kinase, these sites may also be targets for other members of the MAPK family.

In order to verify that all three serine residues were being phosphorylated *in vitro*, we conducted *in vitro* phosphorylation experiments on NEIL1-WT in the presence of non-radiolabeled ATP, and performed LC-MS/MS analysis on the tryptic peptides (S6A Fig). Peptides containing S207, S306, and S61 were identified as phosphorylated with high Xcorr coefficients (S6B Fig). Product ion spectra indicate neutral loss peaks for peptides containing S207, S306, and S61 (S7 Fig). These data confirm that NEIL1 can be phosphorylated by JNK1 *in vitro* at S207, S306, and S61.

To analyze the effect of phosphorylation of NEIL1 by JNK1 on the catalytic activity of the enzyme, we used SP:C and AP:C substrates to measure glycosylase and lyase activities, respectively (Fig 6C). Since JNK1 was present in the reaction mixtures, we incubated the oligonucleotide substrates with JNK1 alone to ensure that this kinase did not interfere with the catalytic function of NEIL1. NEIL1 phosphorylated by JNK1 *in vitro* (pNEIL1) cleaves both SP:C and AP:C substrates in a manner similar to unphosphorylated NEIL1 (Fig 6C). Taken together, these data indicate that JNK1 phosphorylates NEIL1 at three serine residues, but seems to have little effect on the catalytic activity of the enzyme. Similar experiments performed using active c-Abl to phosphorylate the OGG1 DNA glycosylase, revealed that phosphorylation had no effect on OGG1 activity [12].

## Discussion

The accumulation of oxidative DNA damage from both exogenous and endogenous sources have been implicated in the development and progression of several diseases such as cancer, obesity, diabetes, Alzheimer’s disease, and others [55–57]. PTMs modulate the activity of several DNA repair enzymes and are essential for signaling the presence of DNA damage, thereby maintaining the integrity of the human genome [29]. The current investigation is the first report of phosphorylation of the NEIL1 enzyme expressed in human cells. We specifically explored the role of phosphorylation of NEIL1, with the understanding that this enzyme could also be a target of other modifications such as acetylation, methylation, ubiquitylation, and sumoylation, in addition to other PTMs. In this study, we detected three serine residues, S207, S306, and S61 that were phosphorylated in HEK293T cells. Phosphorylation is one of the most abundant modifications since approximately 30% of proteins exist in a phosphorylated state [58, 59]. Yet, one of the biggest challenges in studying the direct effects of phosphorylation on proteins purified from mammalian cells is that only a small portion of the target protein is phosphorylated due to a combination of fractional modification of the protein population and the effect of protein turnover.

Phosphomimetics thus provide a rapid and relatively simple means to address questions directly related to differences in enzyme activity and function *in vitro*. Although all mimetics in this analysis were active, subtle differences in enzyme turnover were observed with the S306E and S61E variants, which displayed a reduced ability to turnover (S4A Fig). S306 is located in the disordered C-terminal domain of NEIL1, which is required for *in vivo* enzyme activity and is thought to interact with and stabilize the ordered core domain of the enzyme [60], in addition to fostering interactions with other proteins [61]. Thus phosphorylation of S306 may play a role in stability, enzyme turnover, and may mediate interactions with other proteins such as PCNA. S61, on the other hand, is located on β-strand 3 one of the two β-strands which flank a loop harboring K54. This lysine residue is highly-conserved and absolutely essential for glycosylase activity [62]. *In silico* mutation of S61 to glutamate indicates that this residue is poised to make stabilizing salt bridge contacts with R46, on the neighboring β-strand 2, which could rigidify the loop containing K54 thereby affecting product release. Assessment of these differences in enzyme turnover needs further scrutiny since different protein preparations could lead to altered reaction conditions, thereby influencing turnover rates. In this study we used enzymes made from a single preparation for all assays including glycosylase and lyase activity assays, EMSAs, and multiple turnover reactions.

Each enzyme in a given pathway can be targeted by multiple kinases dependent upon several factors such as tissue or cell type, stage of the cell-cycle, and protein localization. Thus, the first and perhaps easiest step in identifying a potential kinase involves consideration of a consensus motif. In scrutinizing the NEIL1 sequence, we observed the emergence of a common pattern where each of the three serine residues identified in our MS/MS analysis was immediately followed by a proline (….SP…., S5 Fig). Proline in the +1 position following a target serine or threonine residue, is an absolute requirement for members of the MAP kinase family. However, in addition to this requirement, MAP kinases also possess a docking site downstream of the target residue that impacts their specificity [63]. Given these requirements, several members of the MAPK family could be involved in the phosphorylation of the NEIL1 DNA glycosylase. With the advent of prediction algorithms such as PhosphoNET, the daunting task of testing each potential kinase is minimized. In our case, JNK1 was predicted to be the top candidate kinase for the phosphorylation of S207, S306, and S61 and we showed that JNK1 can indeed associate with NEIL1 and phosphorylate the enzyme *in vitro*. However, it is also possible that other members of MAPK family, such as JNK3 and ERK can also phosphorylate and modulate NEIL1’s activity and this still remains to be determined.

Our current analysis did not reveal phosphopeptides for residues Y263 and S269, reported previously [39]. Factors that could contribute to this observation include differences in cell lines, stage of the cell cycle, and cellular location of the phosphorylation event (*i*.*e*. nuclear or cytoplasmic). Based on kinase predictions from the PhosphoNET server, the kinases most likely to be involved in the phosphorylation of Y263 and S269 are the ITK/ BTK kinases and the mTOR/ FRAP kinases, respectively. ITK and BTK are cytoplasmic tyrosine kinases belonging to the Tec family of kinases [64, 65] whereas the mTOR/FRAP kinase is a member of the phosphoinositide three-kinase-related kinase (PIKK) family that regulates eukaryotic translation in response to growth factors and nutrients [66]. In the current analysis, we focused on determining PTMs of NEIL1 isolated from nuclear extracts, which could be the primary reason for not detecting phosphorylated Y263 and S269 in our preparations. Phosphorylation of NEIL1 at these sites therefore still remains to be further evaluated *in vivo*. In summary, (1) we identified three sites of phosphorylation in the human NEIL1 glycosylase, S207, S306, and S61 using a systematic mass spectrometry approach, (2) we demonstrated that phosphorylation has no significant effect on the enzyme’s DNA binding and enzymatic function using single, double, and triple phosphomimetic mutants, and (3) we identified a kinase that is involved in the phosphorylation of NEIL1 at the three sites identified here. Since phosphorylation at the these sites does not alter the enzyme’s activity *in vitro*, we surmise that this modification could be involved in mediating NEIL1’s interaction with other proteins, in determining subcellular localization of the enzyme, or in modulating function in a particular disease. A database search using the catalogue of somatic mutations in cancer (COSMIC) revealed that S207 when mutated to isoleucine is a confirmed somatic mutation in breast carcinoma [67, 68]. The other two phosphorylation sites do not appear to be associated with any type of cancer, based on the data available to date in COSMIC and cBioPortal. These observations require further scrutiny and open future avenues in studying the effects of phosphorylation on NEIL1 and other DNA glycosylases.

## Supporting Information

S1 Fig Sequence alignments, location of the phosphorylation sites in NEIL1, and purification of phosphomimetics. (TIFF)

S2 Fig The NEIL1-Y263E mutant is catalytically dead. (TIF)

S3 Fig Electromobility shift assays (EMSAs) indicate that all NEIL1 variants bind tightly to DNA. (TIFF)

S4 Fig Activity of NEIL1 under multiple turnover conditions. (TIF)

S5 Fig Sequence of NEIL1 highlighting the sites of phosphorylation. (TIF)

S6 Fig *In vitro* kinase assay and verification of phosphorylation via mass spectrometry analysis. (TIF)

S7 Fig NEIL1 can be phosphorylated *in vitro* by JNK1. (TIF)

S1 File Supporting Information—Methods, References, and Figure Legends. (DOC)
