# Supplementary figures and images for: Phosphorylation Sites Identified in the NEIL1 DNA Glycosylase Are Potential Targets for the JNK1 Kinase
Source: PLoS One. 2016 Aug 12;11(8):e0157860. doi: 10.1371/journal.pone.0157860 (PMC4982613; doi:10.1371/journal.pone.0157860)

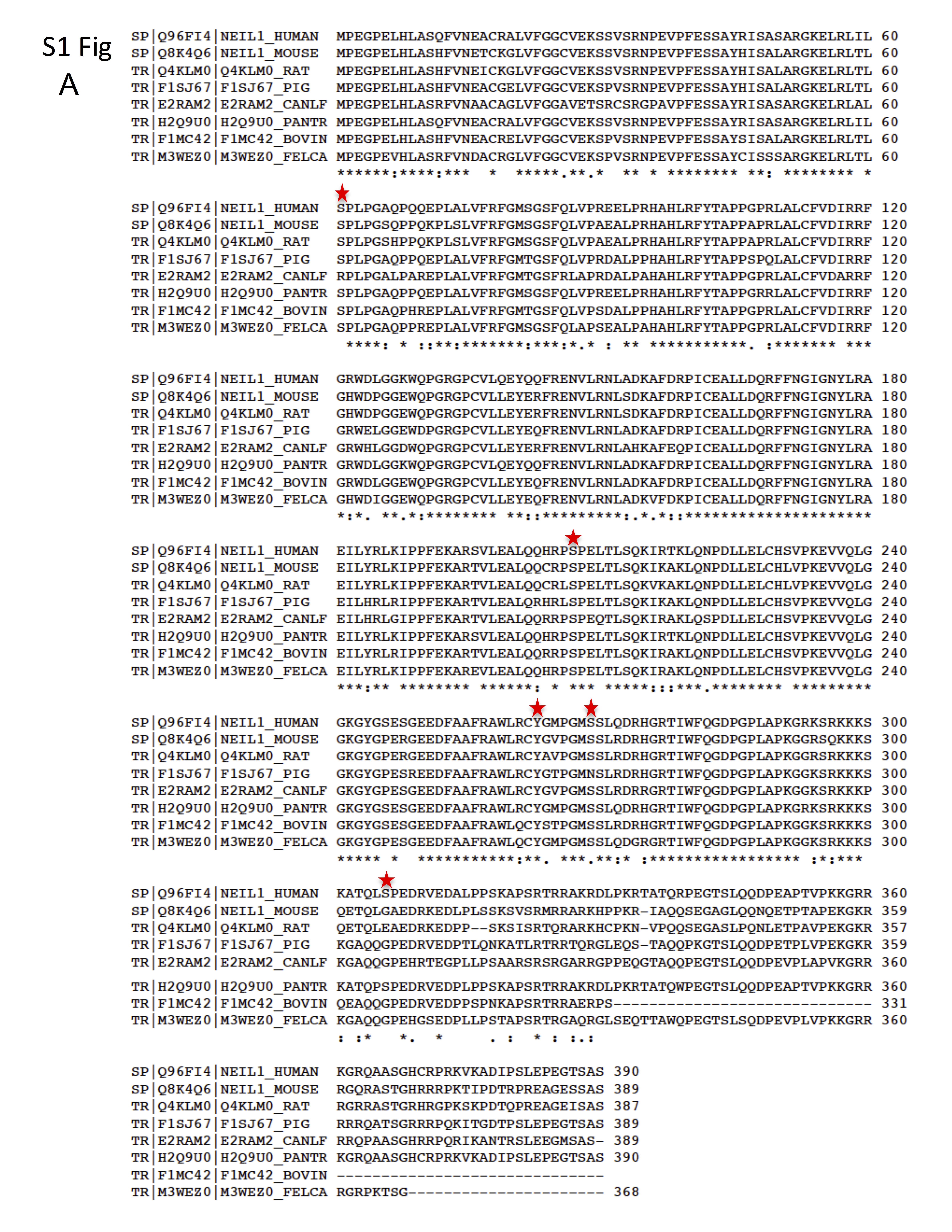

Supplement: S1 Fig — (TIFF) [file pone.0157860.s001.tiff]

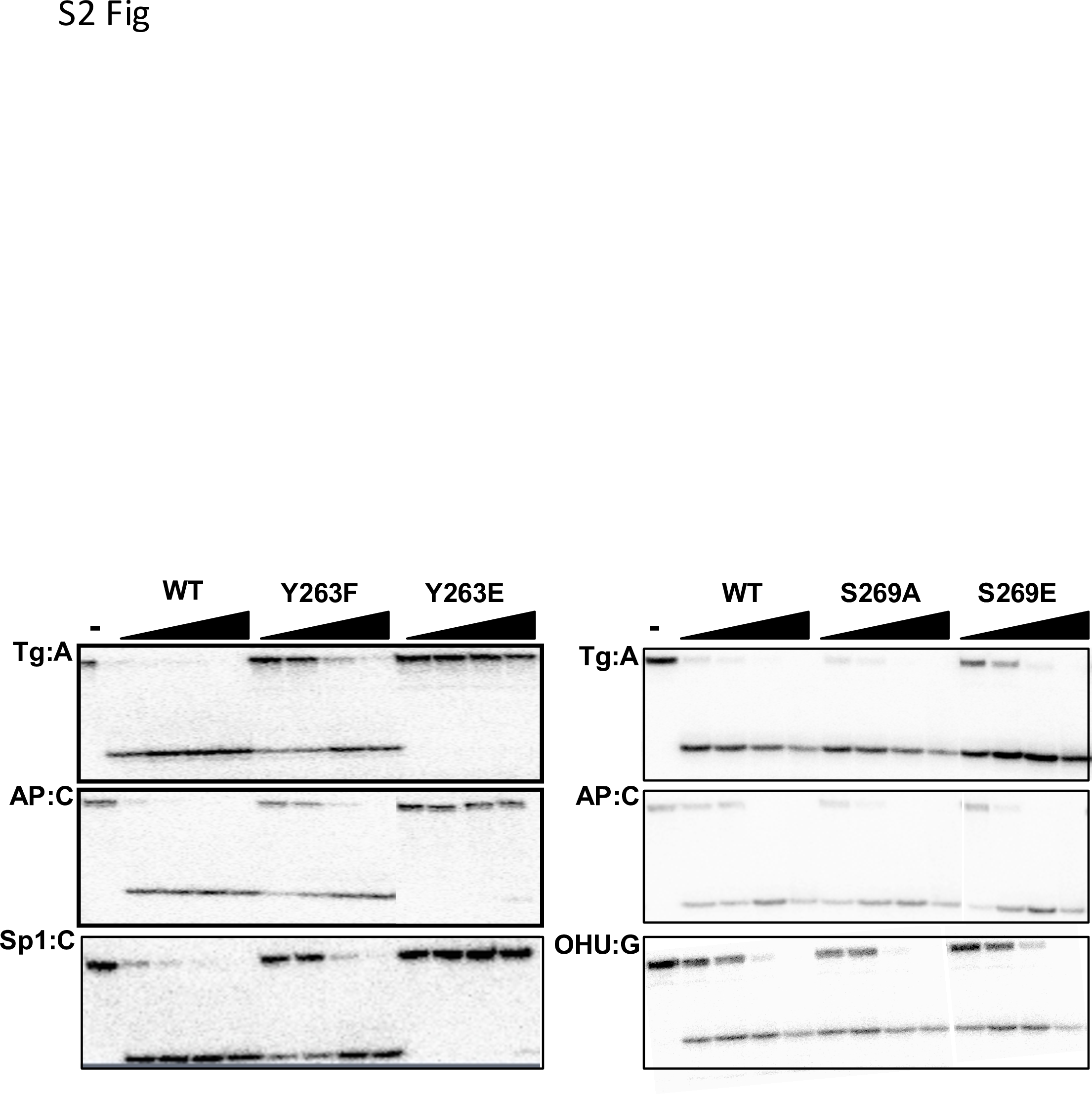

Supplement: S2 Fig — (TIF) [file pone.0157860.s002.tif]

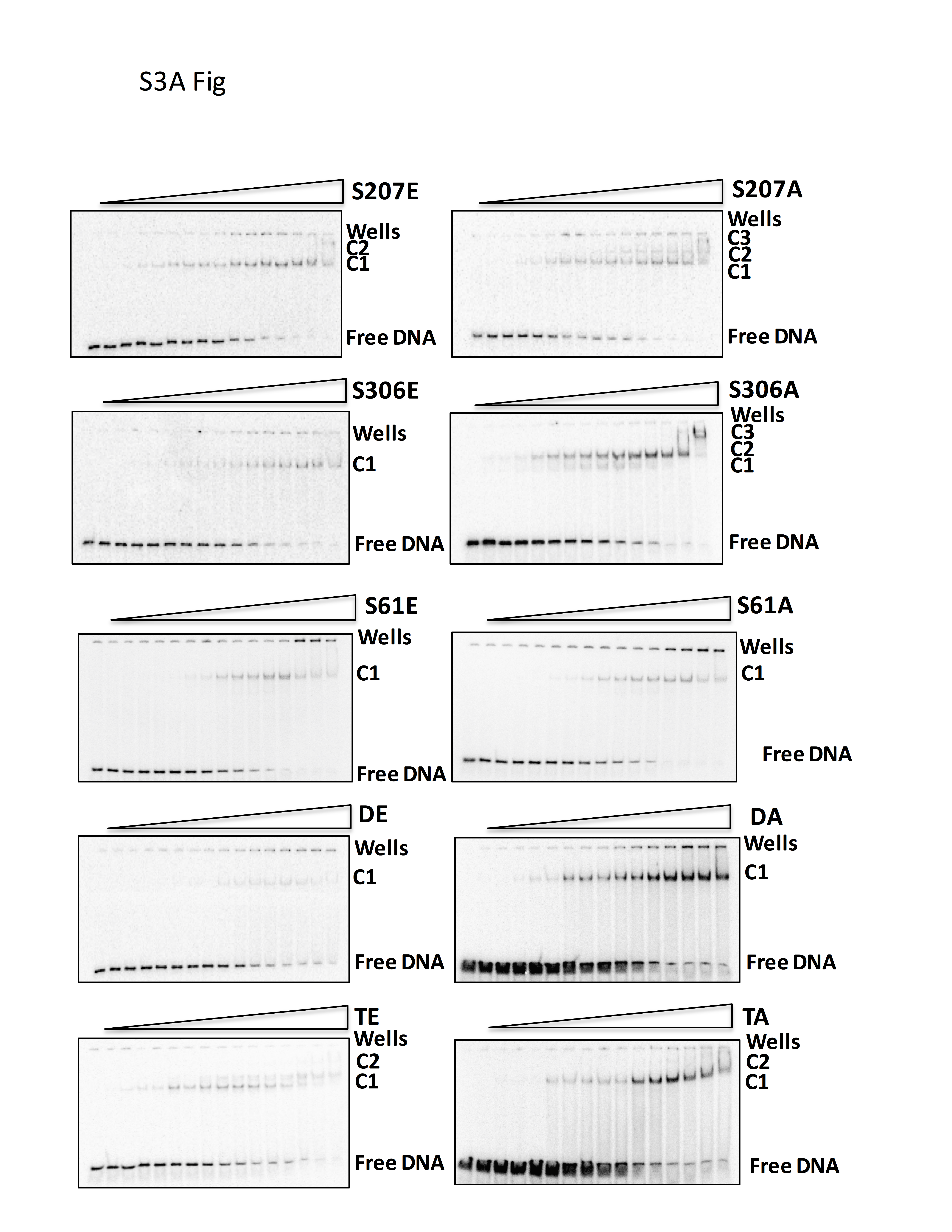

Supplement: S3 Fig — (TIFF) [file pone.0157860.s003.tiff]

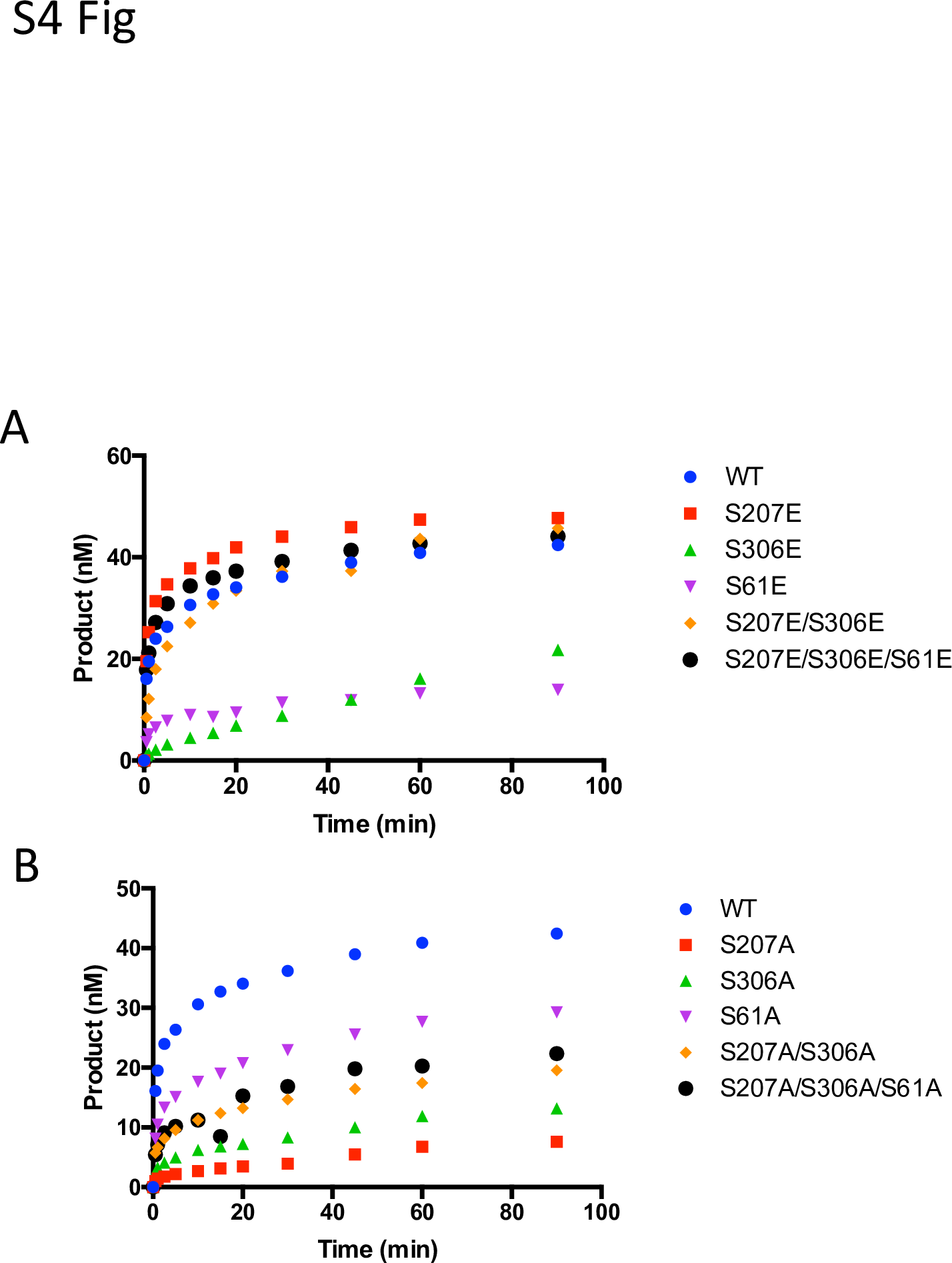

Supplement: S4 Fig — (TIF) [file pone.0157860.s004.tif]

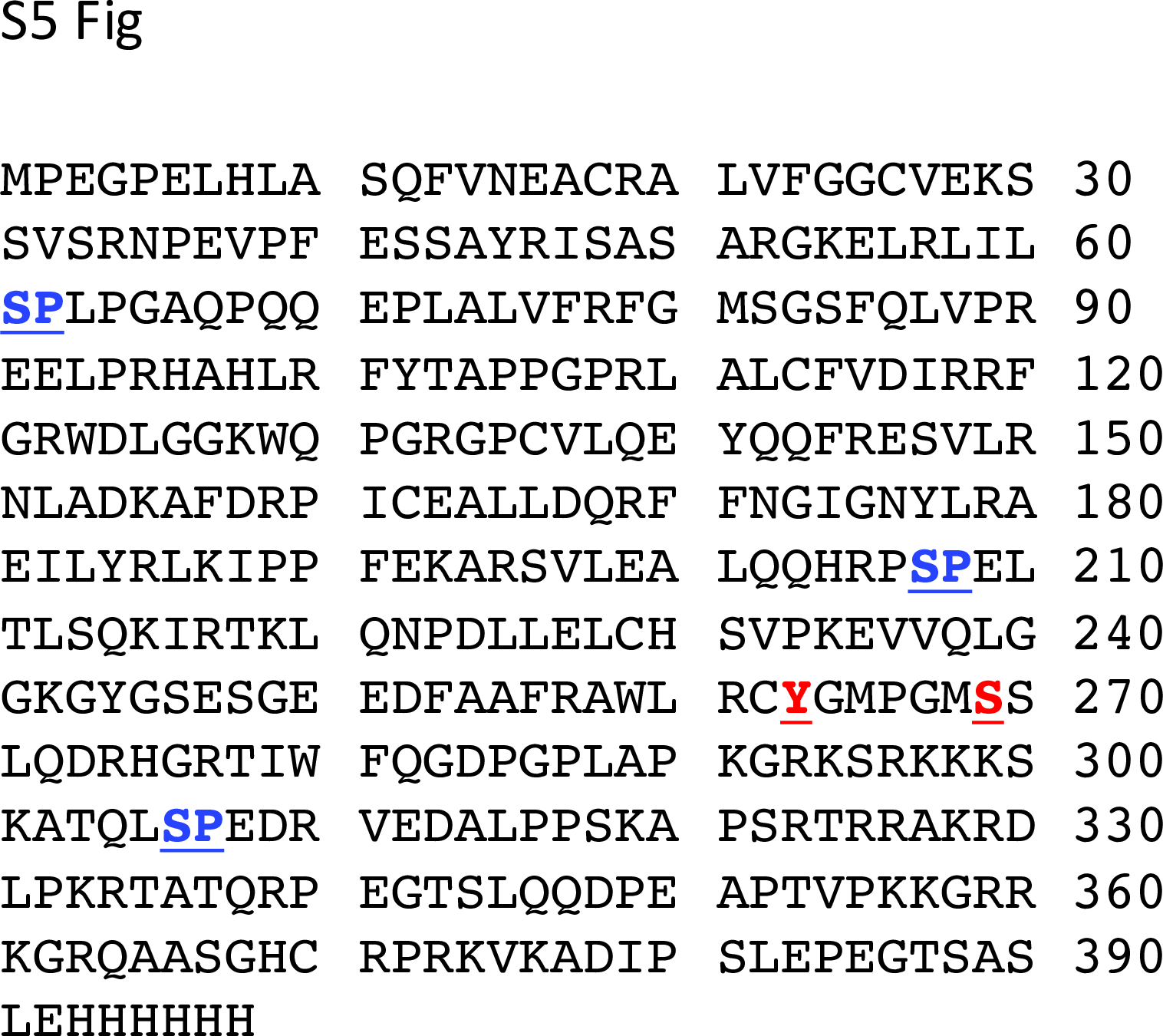

Supplement: S5 Fig — (TIF) [file pone.0157860.s005.tif]

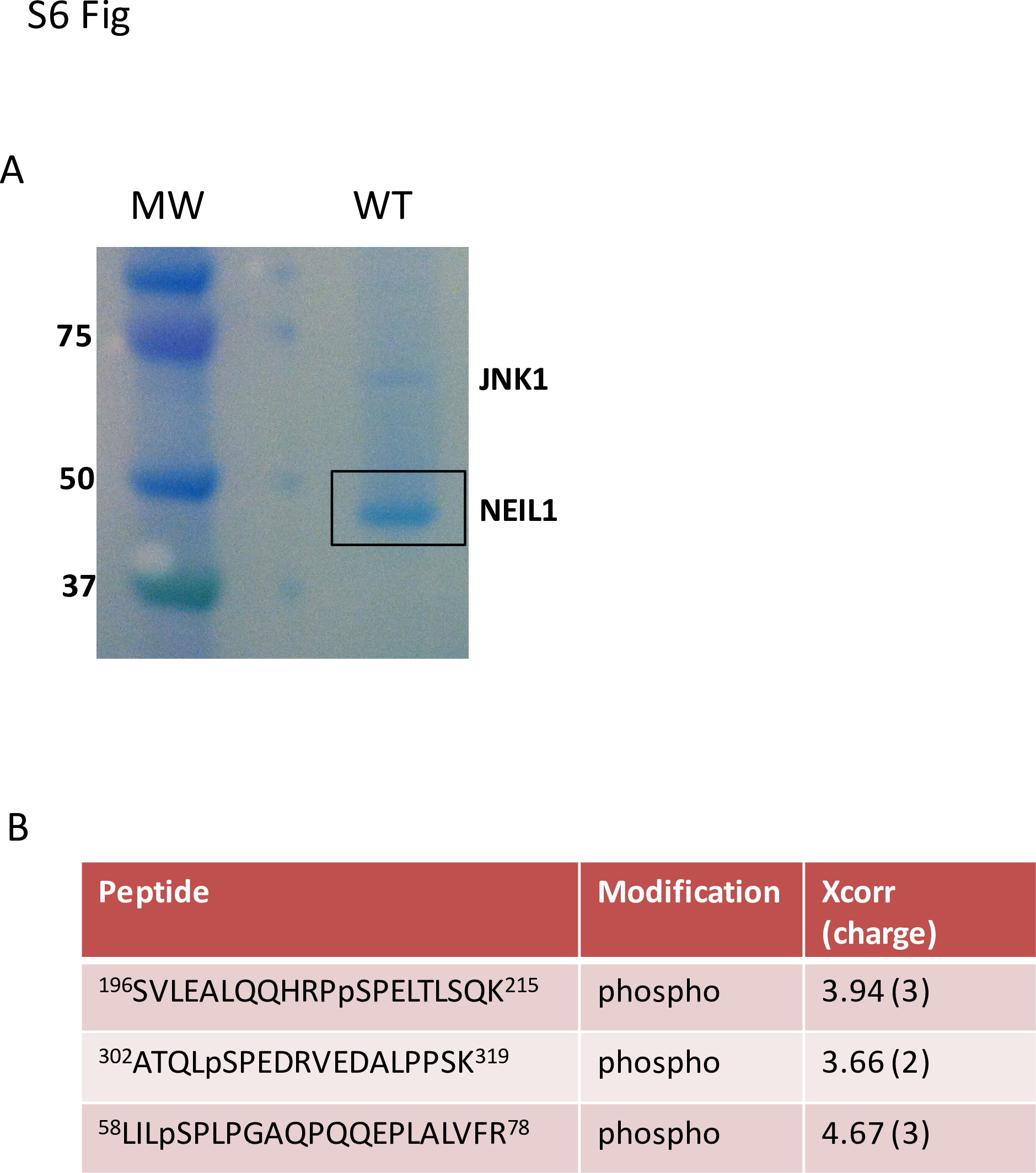

Supplement: S6 Fig — (TIF) [file pone.0157860.s006.tif]

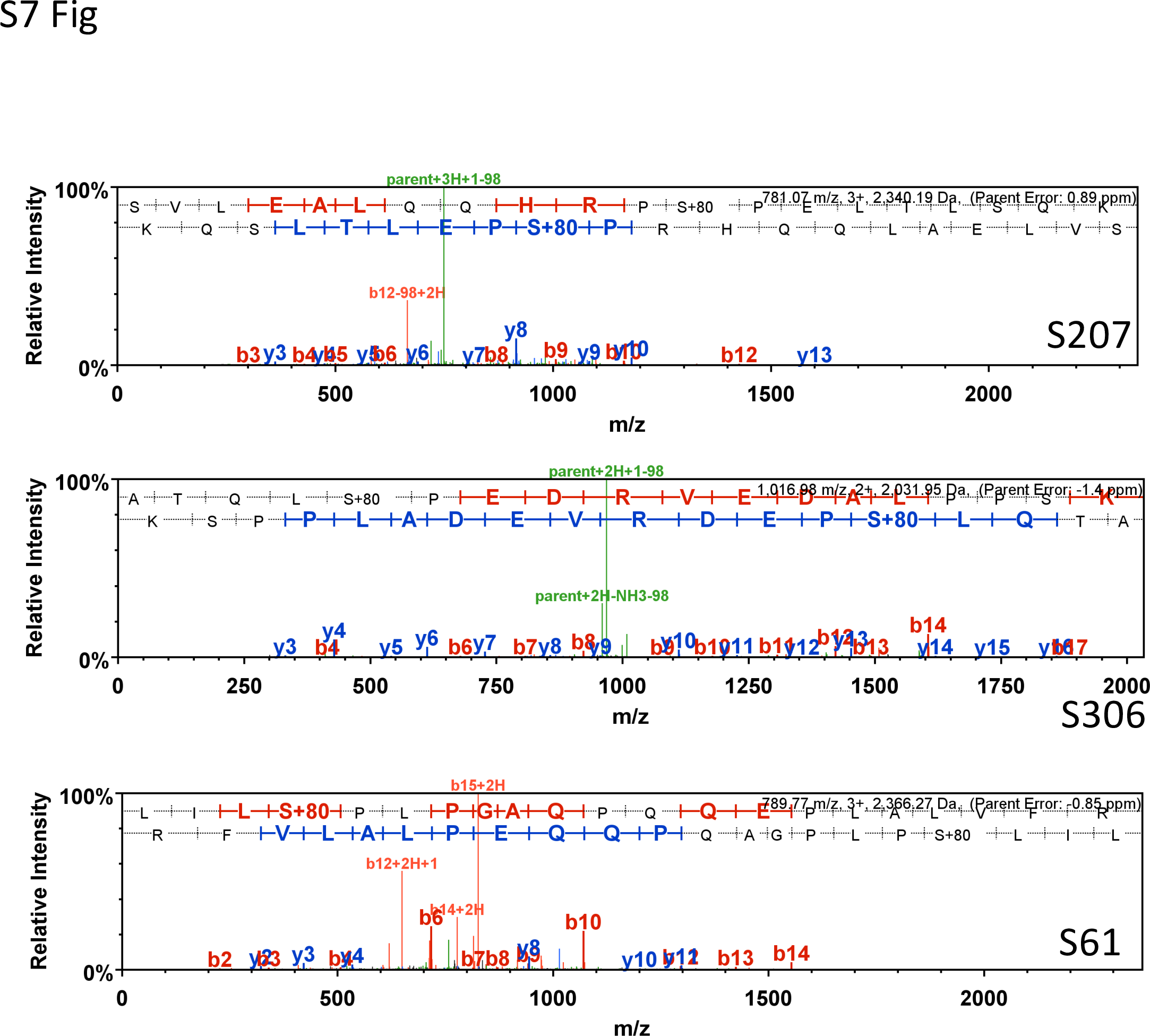

Supplement: S7 Fig — (TIF) [file pone.0157860.s007.tif]
