# Supplementary material for: Phosphorylation Sites Identified in the NEIL1 DNA Glycosylase Are Potential Targets for the JNK1 Kinase
Source: PLoS One. 2016 Aug 12;11(8):e0157860. doi: 10.1371/journal.pone.0157860 (PMC4982613; doi:10.1371/journal.pone.0157860)
Supplement: S1 File — (DOC) [file pone.0157860.s008.doc]

**Phosphorylation sites identified in the NEIL1 DNA glycosylase are potential targets for the JNK1 kinase**

Aishwarya Prakash*^1^, Vy B. Cao, and Sylvie Doublié*

Department of Microbiology and Molecular Genetics, The Markey Center for Molecular Genetics, University of Vermont, Stafford Hall, 95 Carrigan Drive, Burlington, Vermont 05405-0068, United States.

1- Department of Oncologic Sciences, Mitchell Cancer Institute, University of South Alabama, 1660 Springhill Avenue, Mobile, AL, 36604-1405, USA

Tel.: 802-656-9531

Fax: 802-656-8749

E-mail: [Sylvie.Doublie@uvm.edu](mailto:Sylvie.Doublie@uvm.edu)

Tel.: 251-410-4915

E-mail: [aprakash@health.southalabama.edu](../aprakash@health.southalabama.edu)

*To whom correspondence should be addressed.

**Supporting Information**

I. Supporting Methods

II. Supporting Figure Legends

III. Supporting References

**I. Supplemental Methods**

**Multiple turnover assays**

Multiple turnover experiments were performed using the same buffer conditions as the glycosylase assays, with the exception that the substrate was in excess over enzyme ([enzyme] < [substrate]). Assays were performed using 50 nM γ-^32^P labeled Sp:C (2.5% labeled oligonucleotide), incubated with 25 nM total protein. Aliquots were removed at various times (30 sec to 90 min) and quenched by the addition of 10 µL of formamide loading dye. The samples were then run on a 12% denaturing polyacrylamide gel in 1 × TBE at 60 W for 1.5 hr and imaged using an isotope imaging system. Fitting of the data was performed with GraphPad Prism 6.0a software as described previously . Data from multiple-turnover experiments were fitted with equation [1] where *A*_0_ is the amplitude of the burst, *k*_obs_ is rate constant for the burst phase, and *k*_ss_ is the rate of the linear phase.

[P]_t_ = A_0_[1-exp(-k_obs_t)]+k_ss_t [1]

**II. Supplemental Figure Legends**

**S1 Fig*.* Sequence alignments, location of the phosphorylation sites in NEIL1, and purification of phosphomimetics.** (A) Sequence alignments were performed with Clustal O 1.2.1. using NEIL1 sequences from human (Q96FI4), mouse (Q8K4Q6), rat (Q4KLM0), pig (F1SJ67), dog (E2RAM2), chimpanzee (H2Q9U0), cow (F1MC42), and cat (M3WEZ0). All NEIL1 phosphorylation sites are indicated by red stars. (B) The overall structure of human NEIL1 (PDB ID 4NRV ) was superimposed with the crystal structure of its viral ortholog, MvNei1, bound to DNA (PDB ID 3A46 ). A cartoon representation of human NEIL1 is indicated in green and the DNA from the MvNei1-DNA structure is shown in blue and colored by element. The positions of the sites of phosphorylation in NEIL1 are indicated as sticks (colored by element). Two sites, S207 and S306, are in disordered regions of the enzyme, which are indicated by the dashed line. The catalytic P2 residue is also highlighted for orientation. (C) SDS-PAGE gel of NEIL1-WT and all phosphomimetic and phosphoablating variants after purification. 50 pmoles were loaded on the gels for each protein construct.

**S2 Fig*.* The NEIL1-Y263E mutant is catalytically dead.** Glycosylase and lyase activity panel for human NEIL1-WT and the phosphomimetic/ablating mutants for residues Y263 and S269. Glycosylase assays were performed by incubating 20 nM of double-stranded substrates with increasing amounts of enzyme with the following substrate:enzyme ratios: 1:0.5, 1:1, 1:4, and 1:16. “-” indicates no enzyme. Assays were performed at room temperature for 30 minutes using Tg:A, AP:C, and either Sp:C or OHU:G as substrates. Data shown are representative of at least two repeat experiments.

**S3 Fig*.* Electromobility shift assays (EMSAs) indicate that all NEIL1 variants bind tightly to DNA. (A)** EMSA data indicating binding of the phosphomimetic and ablating mutants to ds DNA containing an uncleavable AP site analog (Furan:C) substrate. These assays indicate that the enzymes bind to the Furan:C DNA, as observed by the disappearance of free DNA at increased enzyme concentrations and the appearance of complex, “C1” bands. In some cases, more than 1 complex was observed, as indicated by C2 and C3. At least two repeat experiments were performed for each mutant. (B) Quantification of the EMSAs using the one-site specific binding equation in Prism 6. The data graphed are the mean of two independent experiments.

**S4 Fig. Activity of NEIL1 under multiple turnover conditions.** Multiple-turnover experiments with double-stranded Sp:C were performed where the 50 nM DNA substrate was combined with 25 nM of either WT or phosphomimetic (A) and phosphoablating mutants (B). Aliquots were removed from the reaction at various time points and stopped with formamide loading dye. Data plotted are the mean from two experimental repeats.

**S5 Fig*.* Sequence of NEIL1 highlighting the sites of phosphorylation.** S61, S207, and S306 displayed in blue are all followed by a proline. The proline in the +1 position is an absolute requirement for phosphorylation by the MAP family of kinases. Y263 and S269 are displayed in red and are predicted to be sites of phosphorylation by the ITK/BTK and mTOR/FRAP kinases, respectively (PhosphoNET ).

**S6 Fig*.* *In vitro* kinase assay and verification of phosphorylation via mass spectrometry analysis.** (A) Phosphorylation of NEIL1 by JNK1 using an *in vitro* kinase assay where purified NEIL1-WT was incubated with active JNK1 kinase in the presence of ATP for 30 minutes at 32 °C. The reaction was run on an SDS-PAGE gel stained with GelCode Blue (Pierce). (B) List of phosphorylated peptides identified from LC-MS/MS analysis of pNEIL1 with their corresponding Xcorr values.

**S7 Fig. NEIL1 can be phosphorylated *in vitro* by JNK1.** Product-ion spectra (Scaffold software 4.3) for each phosphorylated peptide are displayed. The b- and y-ions are shown in red and blue, respectively, and the neutral loss peak resulting from the loss of a phosphogroup from the parent ion is indicated in green.

**III. Supplemental References**

1. Krishnamurthy, N., Zhao, X., Burrows, C.J. and David, S.S. (2008) Superior removal of hydantoin lesions relative to other oxidized bases by the human DNA glycosylase hNEIL1. *Biochemistry*, **47**, 7137-7146.

2. Sievers, F., Wilm, A., Dineen, D., Gibson, T.J., Karplus, K., Li, W., Lopez, R., McWilliam, H., Remmert, M., Soding, J. *et al.* (2011) Fast, scalable generation of high-quality protein multiple sequence alignments using Clustal Omega. *Mol Syst Biol*, **7**, 539.

3. Prakash, A., Carroll, B.L., Sweasy, J.B., Wallace, S.S. and Doublie, S. (2014) Genome and cancer single nucleotide polymorphisms of the human NEIL1 DNA glycosylase: activity, structure, and the effect of editing. *DNA Repair (Amst)*, **14**, 17-26.

4. Imamura, K., Wallace, S.S. and Doublie, S. (2009) Structural characterization of a viral NEIL1 ortholog unliganded and bound to abasic site-containing DNA. *J Biol Chem*, **284**, 26174-26183.

5. Safaei, J., Manuch, J., Gupta, A., Stacho, L. and Pelech, S. (2011) Prediction of 492 human protein kinase substrate specificities. *Proteome Sci*, **9 Suppl 1**, S6.
